# How to do no harm: empowering local leaders to make care safer in low-resource settings

**DOI:** 10.1136/archdischild-2020-320631

**Published:** 2021-02-11

**Authors:** Charles A Vincent, Mwanamvua Mboga, David Gathara, Fred Were, Rene Amalberti, Mike English

**Affiliations:** 1 Experimental Psychology, University of Oxford, Oxford, UK; 2 Health Services Unit, KEMRI-Wellcome Trust, Nairobi, Kenya; 3 London School of Hygiene & Tropical Medicine, London, UK; 4 Department of Paediatrics and Child Health, University of Nairobi, Nairobi, Kenya; 5 Foundation for an Industrial Safety Culture, Toulouse, France; 6 Oxford Centre for Global Health Research, Nuffiled Department of Medicine, University of Oxford, Oxford, UK

**Keywords:** health services research, neonatology, nursing care

## Abstract

In a companion paper, we showed how local hospital leaders could assess systems and identify key safety concerns and targets for system improvement. In the present paper, we consider how these leaders might implement practical, low-cost interventions to improve safety. Our focus is on making immediate safety improvements both to directly improve patient care and as a foundation for advancing care in the longer-term. We describe a ‘portfolio’ approach to safety improvement in four broad categories: prioritising critical processes, such as checking drug doses; strengthening the overall system of care, for example, by introducing multiprofessional handovers; control of known risks, such as only using continuous positive airway pressure when appropriate conditions are met; and enhancing detection and response to hazardous situations, such as introducing brief team meetings to identify and respond to immediate threats and challenges. Local clinical leaders and managers face numerous challenges in delivering safe care but, if given sufficient support, they are nevertheless in a position to bring about major improvements. Skills in improving safety and quality should be recognised as equivalent to any other form of (sub)specialty training and as an essential element of any senior clinical or management role. National professional organisations need to promote appropriate education and provide coaching, mentorship and support to local leaders.

**What is already known on this topic?**

- Patient safety is a key goal of the WHO as a central component of high-quality health systems.
- Increasing efforts have been made to improve quality of care in low-resource settings but identifying harms and developing strategies to deliver safe care has been given less attention.

**What this study adds?**

- We describe a ‘portfolio’ approach to safety improvement in four broad categories: prioritising critical processes, improving the organisation of care, control of risks and enhancing responses to hazardous situations that we believe is relevant to low-resource settings.
- We consider how practitioners, especially those in low-resource setting hospitals, might employ these strategies.
- We focus attention on the possible roles of practitioner groups and professional associations as key to advancing patient safety through collaboration and skill development in this field.

## Background

The WHO has made patient safety a priority. In 2012, the Director General, Margaret Chan, pointed out that it was unwise, and potentially unethical, to expand access to care if it was unsafe.^1^ The current Director General stated that ‘it is an indictment on us all for ever tolerating anything less than care that is effective, safe, and people-centred’.^2^ Many international programmes aim to support low-resource settings (LRS) by introducing new treatments and technologies. However, these programmes give comparatively little attention to improving the safety of the underlying health system.

In the first of this two-part series, we showed how local hospital leaders could assess systems, identify key safety concerns and targets for system improvement. By local hospital leaders, we primarily, though not exclusively, refer to health professionals including doctors, pharmacists and nurses who have important ward, department or facility management roles in LRS.^3^ In the present paper, we consider how these leaders might implement practical, low-cost interventions to improve safety. We again use the example of neonatal care to consider some of the challenges and suggest some initial steps for advancing this agenda. However, we believe the principles are applicable to a much wider set of clinical settings.

### Strategies for safer care: how can these challenges be addressed?

In addressing these issues, we first established a small number of core principles to guide our approach. We wanted to ensure that we focused on safety improvements which could be made by local leaders at low-cost both to improve the care given to current patients and as a foundation for advancing care in the longer-term. Our three principles were:

- Improving the safety of care within the current system is the immediate priority.
- Aims should reflect locally identified priorities and be ambitious but achievable.
- Local leaders could draw from established safety concepts and methods, while recognising that approaches may need to be contextualised.

The range of potentially relevant safety and quality interventions is huge and can be bewildering.^4^ To bring some clarity, we group potential interventions into four ‘families’, each consisting of a set of safety interventions which have a similar underlying rationale and purpose. For example, later in the paper, we discuss the need to improve the delivery of continuous positive airway pressure (CPAP) for newborns. This requires making sure key clinical tasks, such as monitoring, are carried out regularly and reliably.^5^ To achieve this however, it may be necessary to strengthen the underlying system by, for instance, clarifying policies and team responsibilities. In addition to this, placing restrictions on when CPAP can be used makes care safer for the whole population of newborns in the unit. Finally, methods to improve the detection and response to threats and hazards provide a further line of defence This ‘portfolio’ approach avoids naïve ‘fixes’ to entrenched safety problems.^4^

### Prioritising safety critical processes

Work pressures and resource shortages in all settings lead to local adaptive routines and departures from core standards.^6^ In time, these adaptations become normalised and people can become blind to basic priorities in care and core safety concerns.^7 8^ In any system under pressure, healthcare leaders therefore need to address a fundamental question. Which processes are safety-critical and what should we prioritise?

In many clinical settings in LRS, it may be simply not feasible to follow all professional guidelines, so decisions must be made about what is a ‘must do’ and what is ‘do if possible’ among a huge number of potential things to do.^9^ Clearly, priorities will differ for each patient, but many will be common to all patients. Examples of ‘must-dos’ in a LRS might include frequent temperature monitoring of a newly admitted preterm baby, ensuring hand hygiene, carefully checking nasogastric tube placements or cross-checking blood products prior to administration.

The team must therefore agree collectively on which clinical processes need to be delivered reliably and expertly and by whom, and which are less critical and can be carried out if time allows or might be safely delegated. Some examples of safety prioritisation are outlined in table 1.

**Table 1:** Examples of safety critical processes and prioritisation on newborn units

Task/work focus	Observations/experience	Prioritisation
Full assessment and documentation of admission by medical and nursing teams.	Documentation tasks regarded as professional norms may be prioritised over clinical needs.	Agree clear rules on elements of documentation and clinical action that are critical and those that can be deferred to a lower workload period.
Cleaning/disinfecting equipment, incubators/cots as part of infection control.	Task commonly informally delegated to mother or unskilled worker with limited supervision. Frequently not completed.	Agree formal delegation of selected tasks and process of supervision and monitoring. Plan training of unskilled staff to standardise practice and maintain safety.
Checking and documenting babies’ vital signs and routine weighing.	Workforce deficits mean recommended frequency rarely achieved. Observations commonly missed, poorly done or sometimes guessed.	Prioritise patient groups by need and illness severity. Develop consistent approach to delegation. Make clear that inaccurate information may be worse than absent information.
Feeding baby prescribed milk volumes through an existing oro/nasogastric tube.	Often informally delegated to mother or unskilled worker. Frequently missed completely at night.	Formalise task sharing or delegation. Train unskilled personnel or family members. Skilled staff check tube position at agreed intervals.
Checking prescribed drug dose calculations during ward rounds.	Inexperienced medical staff may not be familiar with neonatal prescribing. Nurses may be reluctant to challenge doctors.	Agree dose calculation checking as core responsibility of medical team. Empower all team members to identify and correct errors. Recognise this as a positive intervention and learning opportunity.
Administering oral and intravenous drugs.	Time-pressure results in poorly executed procedures and informal delegation to students, unskilled workers and parents.	Identify high-risk medicines and prioritise as professional nursing task. Consider planned delegation for less critical medicines (eg, oral vitamins).

### Strengthening the system

Strengthening the system as a whole is a different approach to improving safety from focussing on specific critical tasks. In this approach, the aim is to support clinical practice more generally by improving the underlying organisation of care and the working conditions of staff. Leaders might, for instance, decide to develop standard operating procedures for cleaning cots and incubators. Structured drug dispensing for Newborn Units (NBUs) and better drug dilution guides would enhance the reliability of these processes and reduce errors.^10^

Improving teamwork and routine communication are high priorities for LRS, as for better-resourced systems.^11 12^ In LRS, however, severe workforce shortages often mean that nurses may not even join medical rounds.^13^ Introducing a brief structured handover between medical and nursing professions would enhance coordination of care; this might incorporate a routine checklist of important issues such as the numbers and skills of staff on duty, any equipment concerns and the number and condition of the most acutely ill patients.^14–16^ Improvements to the working environment are also an important focus. Some are relatively low-cost, such as better storage and organisation of medical records.^10^ Others are more challenging longer-term ambitions such as improving maintenance and upgrading facilities to achieve better hygiene and infection control.^17^ Table 2 shows a number of illustrative interventions mapped to the system components outlined in our accompanying paper.^10^

**Table 2:** Safer systems: illustrative strategies and interventions for a low-resource NBU

System component	Example interventions
Support of families	Provide organised training, with initial supervision and support, so that families can carry out essential caring tasks on the ward and at home. Enhance communication between health professionals and families throughout the care pathway.
Task and technology	Standardise assessment of all patients on admission using structured forms. This can direct staff with limited experience to key issues such as ascertaining the HIV status of a mother. Standardise drug prescription charts. Provide easily read checklists and tables that enable checking of prescriptions.
Staff	Maintain training logs of key competencies such as resuscitation and immediate care of a preterm baby. Explore formal task-sharing with auxiliary staff or other health professional groups such as nutritionists.
Teamwork and culture	Introduce tools such as whiteboards to facilitate team communication, define essential tasks and who is responsible for each task. Establish joint medical and nursing handovers. Use shared medical and nursing records. Work to build a culture that supports everyone to express safety concerns and immediately communicate possible errors while providing clear guidance to junior team members on when and how to escalate a problem to a senior.
Working conditions	Engage with management to improve hand washing facilities and space for staff breaks. Agree with hospital management and NBU team the minimum requirements for ward supplies of disinfectant and cleaning utensils, the cleaning programme and training of staff.
Organisation and governance	Establish and implement clear policies on the qualifications/training required for administering intravenous drugs. Plan staff rotations to avoid sudden relocation of multiple skilled staff or a deluge of students. Agree minimum safety criteria and provide regular reports to hospital management on how often these are breached as part of a case for improved resources/support.

Maintaining staff well-being and morale is particularly important in all systems. Work in any NBU is stressful even in well-resourced settings. In a Kenyan NBU, staff face many additional challenges due to low staffing levels, shortages of essential drugs and equipment and high caseloads.^8^ Their physical and mental health may suffer and, in the longer term, almost all are at risk of burnout.^18^ Staff may suffer acutely if they feel responsible for an error which harms a patient, with consequent impact on both their personal lives and professional lives, and it is critical to provide support in the aftermath of such events.^19^

### Risk control

Risk control is widely used in other high-risk industries. These industries proactively restrict high hazard activities by placing controls on people and practices. Aviation regulators enforce strict rules governing when a pilot can and cannot take off. A storm in Nairobi will result in all flights to Nairobi grounded at their departure airport or diverted to other airports. Examples in healthcare include stipulating that only trained staff can give high-risk medications such as chemotherapy and restricting which drugs can be prescribed in a primary care setting.^20^

Introducing controls and restrictions can be an enormously powerful means of improving the safety of care.^21^ However, in our experience, such controls are seldom formalised in LRS. Instead, it is often implicitly expected that health workers will attempt the impossible and take on tasks for which they are poorly trained and ill prepared. We use the example of initiating CPAP in a sick newborn to illustrate how rules can be designed to reduce risks to patients and prevent serious harm (box 1). Another might be having clear guidance on who should make decisions on when to perform an exchange transfusion for treating severe jaundice and which team members can conduct the procedure. Reducing heroic but unskilled interventions is in the best interests of the population of mothers and babies on the unit and of staff who can experience damaging guilt when things go wrong, even through no fault of theirs.^22^ More widely introducing clear rules on which drug formulations can be issued to NBUs (as discussed in our companion paper) or governing the use of specific antibiotics such as carbapenems or vancomycin to curb emergence of antimicrobial resistance also fall into this category.

Box 1 **Using controls to reduce risk—the case of neonatal CPAP**

- Nasal CPAP requires a sick baby with severe respiratory distress to have tightly fitting nasal prongs fitted and secured, so an air/oxygen mix can be delivered through the nose and maintain a continuous level of distending pressure to the infant lung. The level of inspired oxygen and pressure and other vital signs should be monitored regularly, ideally at 2 hourly intervals, for several days. Too much oxygen may cause harm and sustained pressure can result in pneumothorax, which can be rapidly life threatening. On balance, evidence suggests use of CPAP improves outcomes when conditions permit its careful use.
- Kenyan experts recommend that if CPAP is to be used, then all shifts comprising entire 24-hour periods should have at least one nurse fully trained in its use and that hospitals should have reliable access to pulse oximetry and emergency imaging for newborns. In addition, a clinician trained in use of CPAP should be on-call and available to review babies’ progress all 7 days of the week. If these conditions are not met, then it can be more dangerous for the baby to be initiated on CPAP than to receive simple nasal oxygen.
- In this way, the risk of harm is managed proactively by consensus by providing rules for when it is safe to initiate CPAP, or conversely when it is not even if the baby might benefit from it in a better resourced setting. Further, local discussions might involve considering: (i) the nurse to patient ratios on the NBU at the time the decision is being made and for the next 24 hours; (ii) the level of acuity of all the babies already on the ward; (iii) the availability of a trained clinician and availability of equipment in case of a pneumothorax and (iv) the availability of sufficient monitoring devices, such as pulse oximeters.

CPAP, continuous positive airway pressure.

We emphasise that such policies and restrictions can be agreed by professional groups. They should be seen, not as a restriction of clinical autonomy, but as a sensible and proportionate form of risk management.

### Detecting and responding to threats and hazards

Safety is achieved partly by attempting to prevent errors and also by actively managing the problems and hazards that inevitably arise. This safety strategy, complementary to other approaches, enhances the ability of people, teams and organisations to respond to risks proactively. Red/amber/green operating theatre guidelines and training in crisis management in anaesthesia are examples of building a capacity to respond to hazard into clinical work.^23^ Briefings and debriefings can be used by ward staff, operating theatre teams and healthcare managers to monitor day-to-day threats to safety. For example, briefings carried out by operating theatre teams provide an opportunity to identify and resolve equipment, staffing, or theatre list order problems before a case starts.^24^

Staff in NBU in LRS rarely have time to engage in simple reflection on ‘are we doing the right things in the right way’. Dedicating even small amounts of time to efficient exchanges, through huddles or 15 min sit-down rounds, is an effective and relatively straightforward means of surfacing safety issues on a regular basis.^25^ Huddles are short team discussions targeted at identifying key risks, raising awareness of current problems and action planning for babies or broader challenges on the ward.

These meetings can only be effective if staff feel able to speak up and openly discuss hazards and problems. Keeping quiet about problems is distressing to staff and dangerous for patients.^26^ In some units, experienced nurses may simply amend prescribing errors that they notice, rather than attempt to address the lack of knowledge or care of junior clinical staff.^27^ Mothers, fearing being castigated, may delay bringing problems to the attention of nurses or clinicians until there is a crisis.^28^

An open, no blame, culture of communication is a priority in all settings but especially in organisations where professional, social or cultural conditions create a strong sense of hierarchy. Leaders can play a particularly important role in fostering a willingness to speak up if a patient is at risk by emphasising open discussion of error and system failures.

### The challenges of implementation

We have argued that there are many different avenues to improving safety in LRS and outlined four families of approaches we believe could be implemented by local leaders and their teams with appropriate support. We are fully aware however that implementing such interventions can be challenging in any environment. For instance, the reduction of central line infections in the USA required changes to the organisation of care, the equipment used, simplification of guidelines, engaging local multidisciplinary teams and a staff education programme.^29 30^ Fortunately, there is now considerable experience of the conditions of successful implementation of safety interventions such as checklists.^31–34^ These include: providing training and learning materials even for apparently simple interventions; clear and visible support from senior clinicians and management; identifying champions in each work setting; clarifying roles and responsibilities of each professional group and providing regular supportive feedback on progress.^32^

Setting out a full agenda and programme for supporting local leaders to improve safety in the ways we describe is beyond the scope of this paper. However, we can indicate some initial directions. One valuable first step would be to assemble a core set of resources to support local leaders. These resources would include standard tools and techniques, with examples of application in LRS, that provide the background conceptual understanding and information to help leaders understand their work systems. Creating networks of local teams and leaders who work on similar problems, and who are regularly in touch to compare progress, has been repeatedly found to be important for sustaining safety improvement work.^35 36^ Ideally, existing networks and professional associations can integrate these safety initiatives alongside their existing activities with communications now facilitated by wider access to online meetings and professional peer groups linked by social media.

## Discussion

We have argued in our two papers that improving the safety of care in LRS health systems is both an ethical and clinical imperative. In contrast to most national and international initiatives, we have focused our proposals on local leadership and local priorities. We suggest that the central aim in almost all cases should first be to move towards a stable system with clear clinical priorities and shared team responsibilities. Such an approach can produce immediate improvements in safety and provide a platform for more ambitious, longer-term improvements and innovations. Local clinical leaders and managers face numerous challenges in delivering safe care but, if given sufficient support, they are nevertheless in a position to bring about major improvements and advocate for the resources they need to further improve safety. However, we need to go beyond didactic training on management processes that is the norm to build individuals’ relational skills.^3 37 38^ Such skills are needed, for example, to enable a local leader to create an environment in which junior staff can speak up about errors or to negotiate with a donor and persuade them not to bring new equipment but instead invest in better hand-washing facilities.

Safety analysis and improvement require skills and understanding which can certainly be learnt but at present this is a neglected field in LRS. WHO and other organisations have provided useful formal safety curricula for use in professional education and training. We believe such formal curricula need to be complemented by an accessible library of methods and interventions illustrated by implementation examples from LRS.^5 31 34^ One critical task though is to empower national leaders, possibly through their national professional organisations, so they can offer the coaching, mentorship and support to local leaders that is needed. Where necessary, this may be facilitated by international collaborations. Skills in improving safety and quality should be part of all specialty training and advanced skills in this area recognised as equivalent to any other form of sub-speciality training and as an essential element of any senior clinical or management role. Hospital management, national and international organisations can provide support and guidance but should maintain a focus on strengthening local leadership and local capacity for safety improvement (table 3).

**Table 3:** Actions to support local leaders

Hospital management	Develop and agree with staff local safety policies based on national policies. Support agreed risk control measures to ensure treatments given safely. Only introduce new technologies if staffing, training and resources sufficient to support effective and safe use.
National professional organisations	Develop of context appropriate guidelines and safety policies. Define levels of resources and skill that must be met before facilities advance to a new level. Provide shared resources and support for safety and quality improvement. Create and support of professional networks.
Funders of research in low-resource countries	Include evaluations of patient safety prior to any intervention. Ensure interventions that aim to advance technology supported care include careful evaluation of effects on patient safety. Include funding for strengthening local systems when introducing new technologies or treatments.
International organisations and professional networks	Sharing of experience. Coaching, mentoring and support. Publish and share accounts of safety improvement in low-resource settings.

Improving safety in the short term is a complement to and not a substitute for longer-term action and increased resources. We are emphatically not suggesting that LRS should accept a lower or second-class standard of care. Our proposal is that making care safe is a foundational step in all systems. Advanced forms of treatment or new technologies should, for example, not be introduced until basic, safe neonatal care has been established. In the longer term, with skilled staff and sufficient resources, healthcare systems in LRS can and will achieve global standards. In the short term however, aspirations to leap forward and provide advanced forms of care, sometimes driven by well-meaning partners with short-term funding, may actually be a barrier to developing the local capacity, skills and safe foundations that are needed both now and in the long-term. In Kenya, and other countries, this is consistent with the aim of building from a position of strength stated in policy documents as providing the ‘best standard of care possible’ and ‘highest attainable standard of health’.^39^

A focus on delivering excellent care to the best standard possible, rather than what might be provided in an ideal world, also enables clinical staff and clinical managers to feel a sense of achievement. Conversely, constantly ‘failing’, because of not being able to meet impossible standards, is very demoralising. Leaders engaged in improvement should ensure that staff receive regular feedback on progress and share successes. Achieving safe care in a low-resource setting is a considerable achievement that needs to be both recognised and celebrated.
